# A narrative review on oxytocin at the intersection of sleep, stress, and social behavior

**DOI:** 10.3389/fnins.2026.1745281

**Published:** 2026-04-21

**Authors:** Ida Luisa Boccalaro, Enrico Rillosi, Oscar D. Ramirez-Plascencia, Roberto De Luca, Carrie E. Mahoney

**Affiliations:** Division of Sleep Medicine, Program in Neuroscience, Department of Neurology, Beth Israel Deaconess Medical Center and Harvard Medical School, Boston, MA, United States

**Keywords:** adaptive emotional regulation, circadian rhythms, cognition, emotion, emotional processing, hippocampal-amygdalar circuits, hypothalamic–pituitary–adrenal (HPA) axis activity, impulsivity

## Abstract

Sleep, stress regulation, and circadian rhythms form an interdependent network that shapes cognition, emotion, and social behavior. Disruption of any component can amplify stress sensitivity and impair emotional regulation, leading to neurobehavioral instability. This review discusses evidence from human and animal studies to illustrate how oxytocin (OT) may function at multiple brain regions to modulate sleep regulation, stress physiology, and social interaction. We discuss mechanisms by which sleep deficiency heightens hypothalamic–pituitary–adrenal (HPA) axis activity and stress-related behavioral reactivity and impulsivity, and how OT signaling is thought to counteract these effects by reducing HPA output and stress-induced behavioral responses. Furthermore, converging evidence from preclinical and emerging human studies suggests that OT release may contribute to non-rapid eye movement (NREM) and rapid eye movement (REM) sleep stability potentially via modulation of hippocampal-amygdalar circuits and thalamocortical network activity, including sleep spindle-related dynamics, thereby enhancing emotional processing and social memory. Social isolation, a potent stressor, reduces OT signaling and disrupts sleep–wake dynamics, suggesting a mechanistic link between positive social interaction and sleep maintenance. Collectively, we propose OT as a key neuromodulatory regulator at the intersection of sleep, stress resilience, and social behavior, providing new insights into the neuroendocrine pathways that underlie adaptive emotional regulation and identifying potential therapeutic targets for stress-related sleep disturbances.

## Interaction between sleep, circadian rhythms, stress and social behavior

Sleep, circadian rhythms, and stress regulation constitute an integrated network that is essential for both physiological and psychological stability. Disruption of any component within the sleep-circadian-stress network can negatively influence the others, resulting in impairments in cognition, emotional regulation, mood, and social behavior (Walker et al., 2020; Lo Martire et al., 2020). Adequate sleep is critical for optimal cognitive and emotional functioning (Goldstein and Walker, 2014). Insufficient sleep compromises attention, memory, and decision-making (Zimmerman et al., 2024) and disrupts emotional regulation through alterations in cortico-limbic circuitry (Goldstein and Walker, 2014). Moreover, chronic sleep deficiency, presenting as primary insomnia, fragmented sleep, and/or insufficient sleep duration, elevates stress-related hormones, including cortisol (Lo Martire et al., 2020), increases irritability (Whiting et al., 2023), reduces prosocial behavior and willingness to help others (Simon et al., 2022), and impairs social motivation and subsequent communication (Holding et al., 2019; Palmer et al., 2023). Such disturbances further worsen mood dysregulation and circadian misalignment (Lo Martire et al., 2020).

Beyond sleep duration, sleep stability is a critical determinant of emotional and psychological brain function. Converging evidence from human and animal studies demonstrates mechanistic links between sleep fragmentation, dysfunction within affective neural circuits, and the high prevalence of sleep disturbances observed in psychiatric disorders (Goldstein and Walker, 2014). Gerhardsson et al. (2019) demonstrated that a single night of sleep deprivation significantly reduced accuracy and increased omission errors on an emotional working memory task, irrespective of stimulus valence, while paradoxically accelerating responses to positive stimuli. This pattern suggests disrupted functional coordination between the amygdala and prefrontal cortex (mPFC), leading to impaired sustained attention and compromised decision-making under conditions of emotional load (Gerhardsson et al., 2019). Such disruptions in cortico-limbic connectivity, particularly in regions densely expressing oxytocin receptors (OTRs), are likely to impair the neural substrates underlying adaptive social cognition and effective interpersonal behavior. Accumulating sleep debt has been associated with increased depressive symptoms and social withdrawal (Okajima et al., 2021), suggesting that sleep instability may compromise oxytocin-mediated buffering mechanisms that are critical for maintaining emotional resilience and promoting prosocial behavior. Collectively, these findings suggest that reducing accumulated sleep debt may be a key target for improving workplace wellbeing and productivity (Okajima et al., 2021). Accordingly, the consequences of sleep disruption extend beyond broad cognitive and occupational impairments to include more nuanced yet functionally significant alterations in social cognition and interpersonal functioning. Together, these findings highlight the extensive range of psychological and behavioral domains that are vulnerable to insufficient, fragmented, or unstable sleep (Okajima et al., 2021).

Social behavior is highly sensitive to sleep loss and extends beyond empathy alone to include prosocial motivation, social decision-making, and interpersonal engagement. Even modest, night-to-night reductions in sleep quality decrease individuals’ willingness to help others. Sleep loss disrupts neural systems underlying social valuation, as evidenced by reduced activation in the medial prefrontal cortex (mPFC) and temporoparietal junction (TPJ), regions critically involved in mentalizing and other-oriented decision-making (Simon et al., 2022). Importantly, these alterations in social behavior occur independently of self-reported mood or motivation and are associated with elevated cortisol levels and increased autonomic arousal, suggesting a shift away from OT-mediated prosociality toward stress-driven, self-focused processing (Lo Martire et al., 2020; Simon et al., 2022). Collectively, these findings indicate that sleep loss disrupts neural systems underlying social cognition, valuation of others’ needs, and prosocial decision-making. Beyond social behavior per-se, sleep deprivation further impairs cognitive control and emotional regulation via destabilization of OT-modulated cortico-limbic circuits, that are critical for effectively navigating emotionally salient social environments.

Finally, the interaction between sleep loss and stress appears to be complex and, in some cases, counterintuitive. Bottenheft et al. (2023) reported that acute social stress, though not chronic stress, paradoxically restored performance under conditions of total sleep deprivation, improving multitasking, reaction time, and response inhibition. Although both stress and sleep deprivation elevated autonomic arousal, acute stress alone was associated with improved mood and reduced perceived effort. While these findings were demonstrated in cognitive domains, acute social stress may transiently restore performance during total sleep deprivation by engaging arousal-related neural systems (Bottenheft et al., 2023).

This review aims to synthesize recent evidence linking sleep deficiency, stress, social isolation, and circadian disruption, with particular emphasis on the role of oxytocin (OT) in sleep regulation and social–emotional processes. Specifically, we examine OT’s diverse neurobiological functions and highlight emerging findings implicating OT signaling in the stabilization of non-rapid eye movement (NREM) sleep and in transitions to rapid eye movement (REM) sleep - domains in which mechanistic understanding remains limited. By integrating findings from human, non-human primate, and animal (mouse, rat and voles) studies across sleep, affective, and social neuroscience, this review seeks to elucidate how OT may serve as a mechanistic link between sleep stability and emotional and social functioning. In doing so, we review and identify critical knowledge gaps and outline key directions for future research.

## Oxytocin—the central modulator

OT is widely recognized as a key player in social behavior and modulation of stress responses. Despite OT’s broad effects, in the mammalian brain, only a few thousand hypothalamic neurons, which are primarily located in the paraventricular (PVN) and supraoptic nuclei (SON), synthesize OT (Xi et al., 1999; Ishunina and Swaab, 1999; Jurek and Neumann, 2018; Althammer et al., 2018; Bárez-López et al., 2023). OT is then released into the bloodstream via the posterior pituitary to act hormonally on the uterus and mammary glands (Tsingotjidou, 2022). Branches from the magnocellular and parvocellular neurons of PVN and SON also send projections throughout the brain, where OT is released both synaptically and diffusely (Althammer et al., 2021), functioning as a neuromodulator in regions such as the amygdala, hippocampus, PFC, and bed nucleus of the stria terminalis (BNST), influencing social, emotional, and stress-related behaviors (Tsingotjidou, 2022). Consistent with this distribution, OTRs are widely expressed across these regions throughout the brain, including regions salient to social and emotional regulation such as the limbic, hypothalamic, and PFC areas (Jurek and Neumann, 2018; Bakos et al., 2018). That said, the existing literature positions OT at a critical node uniquely suited to integrate social, emotional, and potentially arousal- and sleep-related processes. Its anatomical and functional connectivity with arousal and stress-regulatory pathways provides plausible mechanisms through which OT may influence sleep regulation, particularly under conditions of chronic stress or social isolation. However, many aspects of these mechanisms remain unresolved. Moreover, findings are often difficult to generalize due to variability across species, physiopathological conditions, developmental stages, and methodological approaches employed across studies (Tabak et al., 2023).

## Oxytocin and social behavior

Experiments in rodent models have established OT’s role in social recognition and affective behavior. OT knockout (OTKO) animals consistently show behavioral deficits such as impaired social investigation and memory (Ferguson et al., 2000). Moreover, OTKO pups vocalize less when separated from their mothers, and OTKO adults display increased aggression compared to wild-type counterparts (Winslow and Insel, 2002). In addition, OTKO mice fail to recognize littermates even after repeated exposures; however, administration of OT restores this social recognition deficit (Ferguson et al., 2000; Neumann, 2008; Lukas et al., 2011). Evidence indicates that prolonged social isolation reduces OT receptor (OTR) expression, without necessarily reducing OT peptide release (Tanaka et al., 2010; Walker et al., 2019). OTRKO models have also provided important mechanistic insights. Similar to OTKO mice, male OTRKO mice exhibit deficits in social recognition, including an inability to remember familiar females (Lee et al., 2008). Additional studies have reported increased autism-like social behaviors in OTRKO mice (Sala et al., 2011) and a reduced preference for social novelty in OTRKO prairie voles (Horie et al., 2019). Furthermore, Burkett et al. (2016) demonstrated that prairie voles display OT-dependent consolation behavior toward stressed conspecifics. This response is characterized by increased partner-directed grooming, emotional contagion (i.e., matching fear and anxiety responses), and physiological state matching, underscoring a critical role for OT signaling in empathy-like social processes. Importantly, pharmacological blockade of OTR signaling abolishes consolation behavior, implicating conserved neural regions across species, including the anterior cingulate and prelimbic cortex. These regions exhibit high OTR expression and are thought to mediate oxytocin-dependent empathy-like processes (Burkett et al., 2016). Peer-relationship formation was delayed and less stable in prairie voles that lack a functional OTR (Black et al., 2025) Calcagnoli et al. (2014) demonstrated that chronic intracerebroventricular infusion of OT via osmotic minipumps enhanced social exploration and reduced aggression in a resident-intruder paradigm. Conversely, infusion of an OTR antagonist increased the initial expression of aggression, without altering its overall duration. Notably, these behavioral effects persisted for up to 7 days following cessation of the infusions (Calcagnoli et al., 2014). Overall, these findings support a role of OT signaling in mediating social interaction and social memory.

## Social behavior and sleep

**Figure 1:**
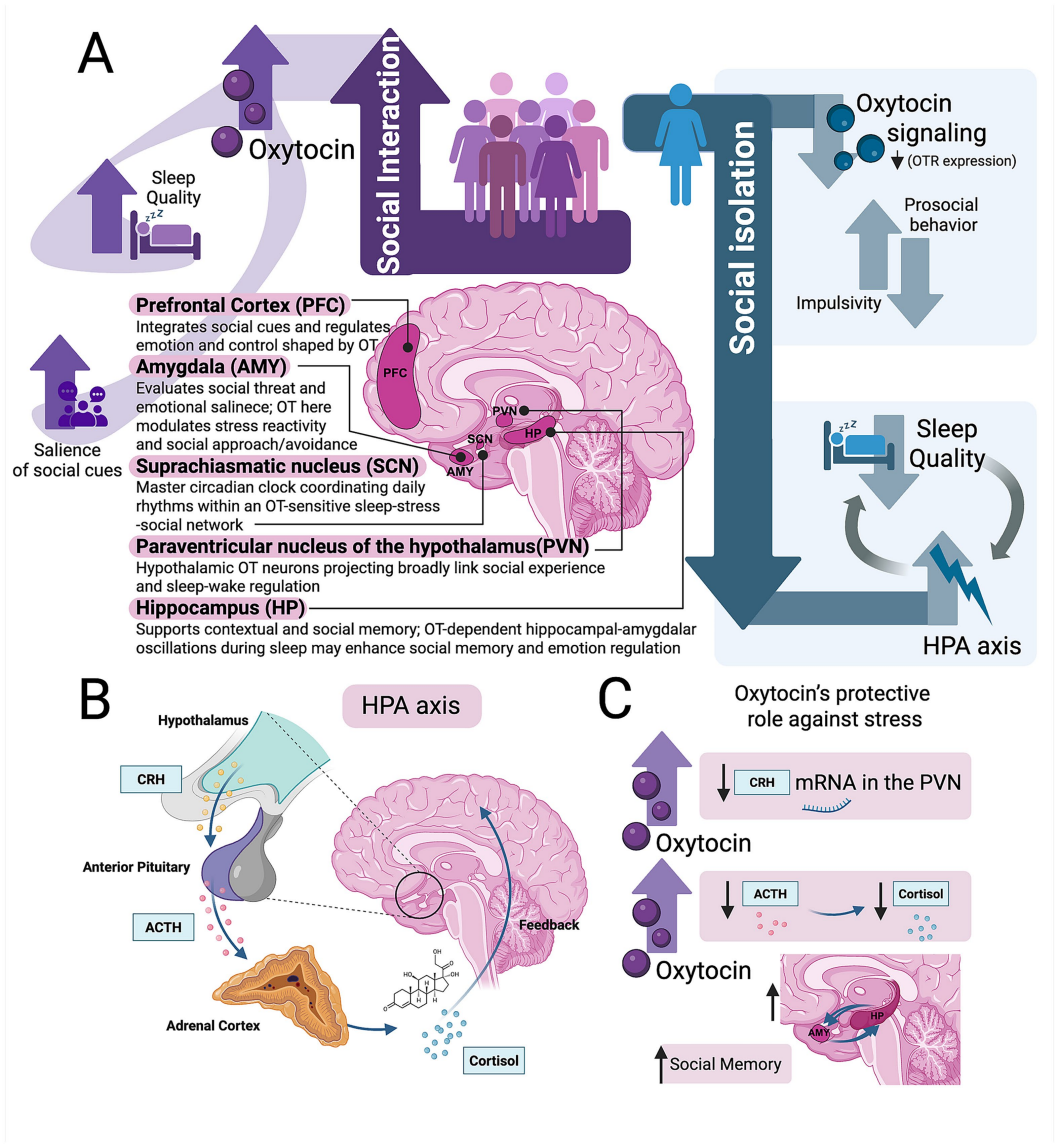
Oxytocin’s modulatory roles in social interaction, sleep, and stress regulation. **(A)** Bidirectional interactions among oxytocin (OT) signaling, sleep, and social behavior occur across key limbic and hypothalamic regions, including the prefrontal cortex (PFC), paraventricular nucleus of the hypothalamus (PVN), suprachiasmatic nucleus (SCN), amygdala (AMY), and hippocampus (HP). Supportive social interactions are associated with increased OT release, which may contribute to improved sleep continuity and amplify the salience of social cues. In contrast, social isolation and chronic sleep loss are linked to reduced OT signaling, increased aggression, and diminished prosocial behavior in animal models, along with increased hypothalamic–pituitary–adrenal (HPA) axis activation and stress sensitivity in both animals and humans. HPA axis overactivation disrupts NREM slow-wave activity and sleep restoration, further compromising emotional regulation and social functioning and potentially creating a feedforward loop among stress, sleep disruption, reduced OT signaling, and maladaptive social behavior. These pathways are proposed to reflect context-dependent and bidirectional interactions rather than uniform causal relationships. **(B)** Schematic representation of the HPA axis. Corticotropin-releasing hormone (CRH) released from the hypothalamic PVN stimulates adrenocorticotropic hormone (ACTH) secretion from the anterior pituitary, leading to glucocorticoid (cortisol) release from the adrenal cortex. Cortisol subsequently induces negative feedback on both the hypothalamus and pituitary to maintain homeostatic balance. **(C)** Oxytocin’s modulatory and protective role in stress regulation. Oxytocin downregulates *Crh* mRNA expression in the PVN, thereby reducing ACTH and cortisol secretion and facilitating physiological stress recovery. In parallel, oxytocin enhances social memory and resilience through modulation of amygdala- and hippocampus-dependent circuits. These mechanisms are primarily supported by preclinical evidence and are thought to contribute to adaptive stress regulation in humans.

Social relationships play a vital role in human health. Social isolation refers to an objective reduction in the size of an individual’s social network and the frequency of social interactions (House et al., 1988; Holt-Lunstad et al., 2010). Individuals who are socially isolated are at increased risk for cardiovascular diseases, cognitive decline, and infectious illnesses (Cohen et al., 1997; Bassuk et al., 1999; Barth et al., 2010). Emerging evidence suggests that the cognitive deterioration associated with social isolation may be mediated, at least in part, by sleep disruption. Loneliness, for example, has been linked to the development of insomnia symptoms in middle-aged and older adults (Qi et al., 2023). Furthermore, individuals exhibit more consolidated sleep and REM sleep when sleeping with a partner (Drews et al., 2020), suggesting that social isolation, or habitual solitary sleep - may negatively impact sleep quality (Figure 1). Interpersonal conflict and relationship strain have been associated with prolonged sleep latency and poorer sleep quality, suggesting that negative social experiences may disrupt sleep architecture in a manner similar to social isolation. More broadly, growing evidence indicates a bidirectional relationship between social isolation (and subsequent loneliness), and sleep behavior. In a large observational and bidirectional Mendelian randomization study, Wang et al. (2024) reported that genetic liability to adverse sleep traits was associated with increased risk of social isolation, supporting a potentially causal pathway from sleep disturbance to social withdrawal. These findings highlight the need to further clarify the directionality and underlying mechanisms linking sleep and social disconnection (Wang et al., 2024).

However, social relationships influence sleep in complex and context-dependent ways. Early mechanistic insights from recent animal work may demonstrate that OT circuits are sensitive to chronic sleep disruption. Wang et al. (2025) showed that chronic sleep deprivation alters neuronal function of PVN^OT^ neurons and that these neurons are necessary for the expression of anxiety-like behaviors induced by sleep loss. Although conducted in rodents and focused on affective outcomes, this study provides causal evidence that sleep disruption engages OT-dependent neural pathways involved in emotional regulation (Wang et al., 2025). Together, these human and animal findings underscore the importance of identifying biological mediators that may link sleep instability with social and emotional dysfunction. Importantly, these observations indicate that social interactions are not uniformly beneficial for sleep. Accordingly, OT’s influence on sleep and social behavior is likely shaped by the valence and context of social interactions rather than reflecting a uniformly prosocial or sleep-promoting effect.

In the following paragraphs, we will examine in greater depth the role of OT in shaping sleep physiology and regulating arousal states, with particular attention to how social context modulates these effects. We will discuss how supportive social environments may buffer stress and promote sleep stability, whereas negative or stressful social experiences can worsen sleep disturbances, potentially through OT-dependent mechanisms. Emerging evidence highlights the need for further investigation into how prosocial and stress-responsive neural circuits regulate sleep, particularly in regions with dense oxytocin receptor (OTR) expression, including the BNST, nucleus accumbens (NAc), prelimbic cortex, PFC, amygdala, and hippocampus.

## Oxytocin and sleep

Whereas the role of OT in social recognition and affiliative behavior is well established, accumulating evidence suggests a previously underappreciated role for OT in the regulation of sleep architecture. This evidence is strongest in animal models, with emerging, but still limited, clinical data in humans. Recent studies indicate that OT may modulate sleep through both direct receptor-mediated signaling and indirect mechanisms, including the release of co-transmitters and interactions with other neuromodulatory systems (Lancel et al., 2003; Bakos et al., 2018; Paletta et al., 2022; Wang et al., 2025). This emerging function is particularly relevant given the ongoing need for novel therapeutic strategies to treat sleep disorders. Currently prescribed hypnotics, including benzodiazepines and Z-drugs, primarily target GABAergic signaling and are associated with significant adverse effects, such as tolerance, dependence, and cognitive impairment (Atkin et al., 2018). Consequently, alternative neurobiological targets are being actively investigated, and OT has emerged as a promising candidate due to its broad physiological and neuromodulatory influence.

In human research, OT is most commonly administered intranasally, typically as single or acute doses ranging from 18 to 40 IU. Short-term, controlled studies generally report no consistent increase in adverse outcomes under standard experimental conditions (MacDonald et al., 2011). However, despite frequent discussion of its therapeutic potential, sleep outcomes are rarely assessed across the broader intranasal OT literature, limiting conclusions regarding its clinical utility for sleep-related endpoints. One of the clearest links between intranasal OT and sleep physiology derives from studies in obstructive sleep apnea (OSA). In one investigation, nocturnal administration of 40 IU intranasal OT was associated with increased total sleep time and improved subjective sleep quality, along with reductions in arousal-associated apnea events (Jain et al., 2017). In a subsequent study, the same group reported that 40 IU intranasal OT reduced the duration of obstructive events and oxygen desaturation without significantly altering overall sleep architecture and without reported adverse effects (Jain et al., 2020). Collectively, these findings provide preliminary support for a role of OT in modulating sleep-disordered breathing. However, conclusions remain limited by small sample sizes, heterogeneous study designs, and the absence of long-term or chronic-dosing data, underscoring the need for larger and more systematic clinical investigations.

Despite growing interest, the reciprocal interactions between neural circuits governing social and emotional processing, and those regulating sleep and arousal remain incompletely understood. Social interactions robustly recruit arousal- and reward-related brain networks, including the ventral tegmental area (VTA), orbitofrontal cortex (OFC), anterior cingulate cortex (ACC), and central amygdala (CeA). These circuits contribute both to the initiation of social contact and to the maintenance of social engagement through processes such as social recognition and cue responsiveness (Rojek-Sito et al., 2023). Importantly, social experience itself can modulate sleep architecture, as demonstrated in both animal and human studies (Meerlo and Turek, 2001; Meerlo et al., 2001; Butt et al., 2015; Kamphuis et al., 2015). Such modulation likely arises through multiple, non-mutually exclusive mechanisms: (1) wake-promoting or arousal-enhancing effects mediated by OT signaling, (2) direct actions of OT on canonical sleep-regulatory regions, and/or (3) indirect influences through interactions with other neuromodulatory systems, including dopaminergic, serotonergic, and corticotropin-releasing hormone (CRH) pathways (Meerlo et al., 2001; Xiao et al., 2018; Froemke and Young, 2021; Yu et al., 2022; Bian et al., 2022; Musardo et al., 2022).

Anatomically, PVN^OT^ neurons project broadly to brain regions known to influence sleep–wake regulation. These regions include the hippocampus, PFC, ventrolateral periaqueductal gray (vlPAG), pedunculopontine tegmental nucleus (PPT), preoptic area (POA), and lateral hypothalamus (LH). In the LH, OTRs are expressed on melanin-concentrating hormone (MCH) neurons which are key regulators of REM sleep and sleep stability (Yao et al., 2012). Additional projections from PVN^OT^ neurons to the VTA, basolateral amygdala (BLA), CeA, and lateral septum (LS) further position OT to influence sleep architecture through modulation of reward processing, emotional salience, and arousal states (Grinevich et al., 2016; Yu et al., 2019; Solié et al., 2022; Wang et al., 2022; Menon et al., 2022; Mahoney et al., 2023). Together, these distributed pathways suggest that OT is anatomically and functionally well situated to integrate social, emotional, and physiological signals relevant to sleep regulation.

In animal models, translational inference is further complicated by variability in OT delivery routes (e.g., intraperitoneal, intracerebral, intranasal), as route-dependent pharmacokinetics can yield qualitatively distinct sleep–wake outcomes. For example, in rats, intraperitoneal OT promoted quiet wakefulness while suppressing active wakefulness, NREM, and REM sleep, with several of these effects mediated via OTR signaling (Raymond et al., 2023). In contrast, intranasal OT produced minimal changes across most sleep–wake parameters at the doses tested. These route-dependent differences underscore the need to steer away from overly simplistic descriptions of the benefits of OT on sleep. More likely, its effects depend on delivery method, dose, circadian timing (e.g., relative to lights-on/lights-off), and whether administration is acute or repeated – paralleling the context-dependent effects of OT on sociability and stress responsiveness.

Beyond its established role in social behavior, OT-mediated modulation of sleep may serve adaptive functions, potentially facilitating the consolidation of social memory and stabilizing emotional reactivity. Elucidating how OT coordinates activity across social and sleep-regulatory neural circuits may therefore provide critical insights into the reciprocal relationships among social experience, emotional regulation, and sleep health.

## Sleep and emotional reactivity—how oxytocin may help to restore balance

Building on the emerging role of OT in sleep regulation, sleep itself is a critical determinant of emotional reactivity and affective stability. Using electroencephalography (EEG) and functional neuroimaging, van der Helm et al. (2011) demonstrated that sleep, particularly REM sleep, attenuates amygdala reactivity to previously encountered emotional stimuli, underscoring a central role for sleep in dampening emotional hyperreactivity (van der Helm et al., 2011). Consistent with these findings, Gujar et al. (2011) reported reduced behavioral and neural responses to adverse stimuli following REM-rich sleep (Gujar et al., 2011). Together, these studies position REM sleep as a key mechanism through which emotional experiences are recalibrated and affective tone is restored.

OT may contribute to this process by modulating neural dynamics within emotion- and memory-related circuits. Experimental evidence indicates that OT promotes theta rhythm generation through the activation of neurons in PFC, hippocampus, amygdala, and LS, thereby enhancing signal-to-noise communication within and across these regions (Tirko et al., 2018; Castagno et al., 2024; Alaerts et al., 2024). Of particular relevance are OT projections to the CeA, where OT-sensitive neurons suppress fear and anxiety-like behaviors in rodent models of aversive conditioning. Social interactions that recruit these OT-responsive CeA neurons may also influence sleep, as CeA activity has been implicated in the modulation of REM sleep duration (Sanford et al., 2002; Wellman et al., 2022). If OT regulates the duration or quality of specific sleep states, it may directly shape emotional regulation, given that prolonged REM sleep following trauma has been associated with reduced emotional reactivity and improved affective processing (van der Helm et al., 2011; Glosemeyer et al., 2020).

Stress-responsive, sleep-regulating circuits may represent an additional point of convergence among OT signaling, sleep regulation, and emotional processing. PVN^OT^ neurons project to LH, which contains key cell populations involved in sleep–wake control. Melanin-concentrating hormone (MCH) neurons, which promote REM sleep, express OTRs and thus could represent a direct substrate through which OT may facilitate REM sleep (Yao et al., 2012). In contrast, wake-promoting orexin/hypocretin neurons express the vasopressin receptor AVPR1A, which can bind OT at higher concentrations (Tsunematsu et al., 2008). Activation of orexin/hypocretin neurons promotes arousal, suppresses sleep, and contributes to the consolidation of behavioral state (De Luca et al., 2022). Notably, at peak concentrations, OT can recruit these neurons during social interactions, suggesting a mechanism through which socially salient stimuli may transiently enhance wakefulness and arousal. Another potential node through which OT may influence sleep and arousal, lies in the midbrain. Yu et al. (2022) identified a subpopulation of VTA neurons that co-release somatostatin (SST) and GABA and are required for sleep promotion following intruder stress (Yu et al., 2022). These neurons may reduce arousal in part by inhibiting wake-promoting orexin/hypocretin neurons in the LH. Whether OT contributes to the recruitment of these VTA SST/GABA neurons after stress, and whether this pathway is engaged by PVN^OT^ projections, remains an important open question.

Recent studies further support a role for OT in sleep-dependent emotional regulation. Chronic sleep deprivation in mice increases anxiety-like behavior and suppresses PVN^OT^ neuron activity (Wang et al., 2025). Conversely, REM-sleep-specific OT release from PVN^OT^ neurons in the PFC rescues social memory deficits induced by chronic sleep deprivation (Liu et al., 2025). These findings suggest that OT-mediated modulation of theta-generating regions (including the hippocampus, amygdala, and PFC) depends on both OTR expression patterns and established PVN^OT^ projection architecture (Grinevich et al., 2016; Liu et al., 2025).

Collectively, this anatomical and receptor-level organization suggests that OT may exert bidirectional control over arousal states: enhancing wakefulness via orexin signaling under certain conditions, while promoting sleep and REM stability through modulation of MCH neurons and stress-responsive circuits under others.

## Interaction of oxytocin and stress in sleep modulation

**Figure 2:**
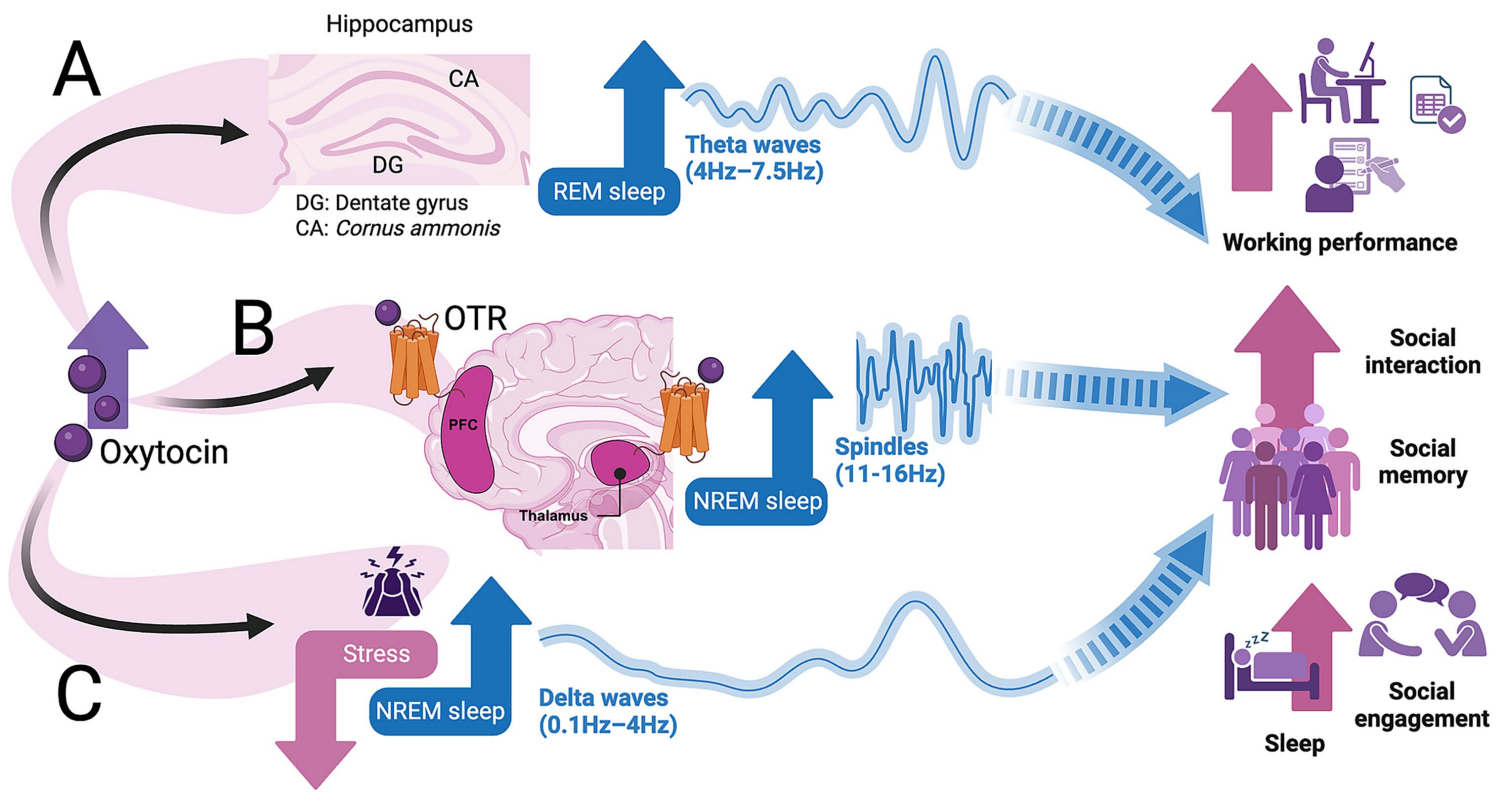
Proposed mechanism of oxytocin’s modulation of sleep and neural oscillations. **(A)** In rodent models, oxytocin enhances synchrony between hippocampal neurons (including CA2 pyramidal neurons and CA1 interneurons) and amygdalar networks, which may contribute to the stabilization of theta oscillations (4–7.5 Hz) associated with rapid eye movement (REM) sleep continuity, memory processing, and transitions between behavioral states. **(B)** Oxytocin signaling through oxytocin receptors (OTRs) expressed in PFC and thalamus may promote spindle activity (11–16 Hz). In rodent studies, oxytocin modulates the excitatory-inhibitory balance within thalamocortical circuits implicated in spindle generation, potentially enhancing sensory gating and sleep-dependent memory consolidation. **(C)** Oxytocin release attenuates stress-related neural activity and HPA axis output in rodent models and modulates stress physiology in humans, contributing to the restoration of homeostatic equilibrium. This modulation may, in turn, enhance non-rapid eye movement (NREM) sleep stability, increase delta oscillations (0.1–4 Hz), and reduce arousal-driven disruptions, thereby supporting restorative sleep and social engagement. Collectively, these mechanisms are hypothesized to exert integrated effects ranging from improved cognitive performance and emotional regulation to enhanced social interaction and overall sleep quality in humans. However, many aspects of these mechanisms remain unresolved. Solid lines = associations or mechanisms supported by experimental evidence. Dashed lines = hypothesized or extrapolated links.

Beyond its direct effects on sleep and emotional circuitry, OT interacts closely with the stress axis and may influence sleep architecture, particularly under conditions of emotional challenge. Both classical and more recent studies have demonstrated robust diurnal rhythms in glucocorticoid secretion, with cortisol levels rising during the late night and early morning - periods that often coincide with REM-rich sleep. This temporal alignment is thought to facilitate the consolidation of emotionally salient memories (Roozendaal et al., 1999; Buchanan and Lovallo, 2001; Payne and Nadel, 2004; Born and Wagner, 2004). Consistent with this framework, administration of hydrocortisone during sleep selectively enhances memory for emotional stimuli while reducing amygdala reactivity, suggesting that glucocorticoid signaling contributes to REM sleep-dependent emotional memory processing (Figure 2A). However, under conditions of chronic stress or social isolation, the coordinated regulation of glucocorticoid secretion and sleep becomes disrupted (van Marle et al., 2013).

Excessive activation of corticotropin-releasing hormone (CRH) signaling has been associated with reductions in slow-wave activity and impaired sleep restoration. For instance, pharmacological manipulation of the stress axis in individuals with post-traumatic stress disorder (PTSD) has been linked to decreased EEG delta power, consistent with diminished slow-wave sleep integrity (Inslicht et al., 2018). In this context, OT may exert a protective effect by dampening central CRH expression and helping to restore sleep stability. Supporting this framework, metyrapone administration in individuals with PTSD increases adrenocorticotropic hormone (ACTH) levels while reducing EEG delta power, suggesting that elevated central CRH drive contributes to impaired slow-wave sleep and reduces restorative function (Inslicht et al., 2018). By attenuating stress-induced arousal and facilitating slow oscillatory activity, OT may therefore counteract stress-related disruptions of sleep architecture and promote more adaptive recovery processes.

Beyond the interaction with the stress network, OT may directly influence sleep architecture. OTRs are expressed in the thalamus, PFC, amygdala, and hippocampus (Jurek and Neumann, 2018; Bakos et al., 2018). These regions are involved with the generation and coordination of sleep spindles (Alaerts et al., 2024). Although Alaerts et al. (2024) did not directly assess sleep outcomes, their findings indicate that OT’s effects on arousal, stress buffering, and emotion regulation could indirectly influence sleep onset and quality (Alaerts et al., 2024). Because OT acts across the aforementioned brain regions, it may modulate thalamocortical circuitry and influence spindle activity. This is particularly relevant under conditions of stress, as spindle dynamics are altered in PTSD. Individuals with PTSD exhibit increased N2 spindle density following exposure to stressful stimuli, and this increase correlates with enhanced recollection of negative memories (Natraj et al., 2023). These findings suggest that stress may bias spindle-related mechanisms toward the maladaptive consolidation of emotionally salient experiences. Collectively, these data position OT as a neuromodulator capable of stabilizing sleep and shaping emotional memory through coordinated effects on the stress axis and thalamocortical oscillatory activity (Figures 2B,C).

Superimposed on socially induced OT release is an additional layer of circadian regulation. The body’s master pacemaker, the suprachiasmatic nucleus (SCN), maintains daily rhythms and entrains physiological processes to environmental light–dark cues (Reppert and Weaver, 2002). In rodents, OT mRNA expression in the PVN exhibits robust circadian oscillations, peaking during the light phase when SCN neuronal activity is elevated (Van Drunen et al., 2024). Disruption of SCN vasoactive intestinal peptide (VIP) signaling prevents activation of PVN^OT^ neurons (Kennett et al., 2008), while dysregulation of intrinsic clock gene expression (i.e., Bmal1) within these neurons flattens rhythmic OT production and release (Van Drunen et al., 2024). Moreover, electrical or optogenetic stimulation of the SCN increases PVN^OT^ neuron excitability (Hermes and Renaud, 1993; Santoso et al., 2018), likely via VIP-mediated signaling (Stangerup and Hannibal, 2020). AVP released from the SCN also appears to directly modulate PVN^OT^ activity (Santoso et al., 2018). Collectively, these findings indicate that the SCN drives rhythmic OT production in the PVN, aligning OT-mediated sleep regulation and social memory processes with the circadian light–dark cycle. Disruption of SCN signaling or PVN clock gene rhythmicity may therefore blunt OT release, weakening both sleep-dependent and circadian modulation of social memory formation.

Consistent with animal studies demonstrating circadian regulation of OT production and release, recent human data also support diurnal variation in endogenous OT levels. In repeated daytime assessments of premenopausal women, salivary OT concentrations increased from morning to early afternoon, indicating time-of-day-dependent fluctuations (Teixeira de Almeida et al., 2025). Extending these findings to youth populations, a study of adolescent psychiatric inpatients and healthy peers reported a consistent diurnal rhythm in salivary OT across two consecutive days, with levels rising toward the evening. Patients exhibited higher overall OT concentrations compared to controls, and diurnal OT levels were associated with measures of emotion regulation and anxiety. Together, these findings suggest that endogenous OT exhibits circadian patterning in humans across developmental stages and may represent a potential biomarker relevant to mental health (Gizuterman et al., 2025).

Future research should clarify how social interactions facilitate central OT release and how OT, in turn, modulates sleep following both positive and negative social experiences. In particular, studies should test whether OT signaling stabilizes sleep after stress and elucidate the mechanisms through which such stabilization occurs (Figure 1A). Anatomical evidence indicates substantial overlap between OTR expression and the distribution of glucocorticoid receptors (GR) and CRH receptors (CRHR) across multiple brain regions that regulate both stress responsiveness and sleep–wake control (Bale and Vale, 2004; Mitre et al., 2016).

These observations raise several key questions: (1) Does reduced social touch or social interaction diminish the sensitivity of relevant circuits to OT? (2) Are stress-related sleep alterations driven primarily by reduced OT signaling, heightened stress reactivity, or their interaction?

## Oxytocin and stress

OT modulates anxiety and stress responses in both animals and humans through multiple peripheral and central mechanisms (Landgraf et al., 1982; Grippo et al., 2007; Grippo et al., 2009; Neumann and Landgraf, 2012; Babygirija et al., 2012; Jamieson et al., 2017; Jurek and Neumann, 2018). At the autonomic level, sympathetic markers such as blood pressure and heart rate are regulated by PVN^OT^ neurons (Zhang et al., 2025). Centrally, stress-induced OT release, both within the brain and peripherally, attenuates hypothalamic–pituitary–adrenal (HPA) axis output by modulating CRH neuron activity (Figure 1B) (Jamieson et al., 2017; Pati et al., 2022). In turn, OTKO animals exhibit heightened corticosterone responses following psychogenic stress compared to wild-type controls (Amico et al., 2008). In humans, associations between endogenous OT levels, stress reactivity, and mood regulation have also been reported, although substantial interindividual variability exists (Kirsch, 2015; Raymond et al., 2025). Together, these findings support a role for PVN^OT^ neurons in coordinating cardiovascular and neuroendocrine stress responses across both physiological and pathological conditions.

Acute and chronic sleep deficiency are increasingly prevalent in modern society and have well-documented consequences for emotional regulation, stress sensitivity, and cognitive performance (Tomaso et al., 2021; Ochab et al., 2021; Demichelis et al., 2022). Experimental and meta-analytic evidence indicates that even moderate sleep loss increases subjective stress and aggressive behavior while impairing self-control, decision-making, and cognitive flexibility (Saksvik-Lehouillier et al., 2020; Demichelis et al., 2022). In adolescents, alterations in cortico-striatal connectivity have been shown to underlie the bidirectional relationship between sleep duration and impulsivity, underscoring increased neurodevelopmental vulnerability to insufficient sleep (Yang et al., 2023).

Many of these behavioral alterations appear to be mediated, at least in part, by dysregulation of the HPA axis. In humans, acute sleep deprivation elevates baseline cortisol levels and amplifies cortisol responses to psychosocial stressors, consistent with a sensitized stress response (Minkel et al., 2014). In contrast, rodent models of prolonged sleep loss demonstrate a blunted ACTH response to stress despite preserved corticosterone secretion, suggesting altered pituitary sensitivity and longer-term neuroendocrine adaptations (Meerlo et al., 2002; Engeland and Arnhold, 2005; Novati et al., 2008). Such HPA axis dysregulation likely contributes to emotional instability, impaired cognitive control, and increased vulnerability to stress-related psychopathology, with accumulating evidence indicating sex- and age-dependent differences in sensitivity to sleep loss (Murack et al., 2021; Stanton et al., 2023).

Sleep-stage specific regulation of stress hormones further highlights the close coupling between endocrine signaling, sleep architecture, and memory processing. NREM sleep that occurs early in the night is characterized by a suppression of ACTH and cortisol secretion, a process that facilitates hippocampal-dependent memory consolidation (Späth-Schwalbe et al., 1993; Plihal and Born, 1999). Pharmacological reduction of cortisol using dexamethasone enhances NREM slow-wave activity in humans, reinforcing the role of glucocorticoids in shaping sleep depth and restorative function (de Feijter et al., 2022).

Within this context, OT attenuates excessive HPA axis activation by inhibiting *Crh* gene transcription in the PVN (Figure 1C), thereby reducing downstream ACTH and glucocorticoid release and promoting recovery from acute stress (Lightman and Young, 1989; Jamieson et al., 2017). These effects are mediated, in part, through GABAergic inhibition of CRH neurons and associated intracellular signaling cascades. In models of chronic stress or glucocorticoid excess, peripheral OT administration, rather than direct central action on the HPA axis, has been shown to prevent adrenal atrophy and restore catecholamine responses to acute stress, thereby enhancing stress resilience (Stanić et al., 2017; Chaipunko et al., 2024). OT also modulates amygdala-hippocampal circuitry and serotonergic tone, contributing to emotional regulation and potentially mitigating affect-driven impulsivity (Lan et al., 2022). Although direct studies examining OT in the context of sleep deficiency remain limited, its well-established role in stress regulation suggests that OT may help counteract the heightened stress sensitivity and impulsivity associated with insufficient sleep (Figure 1).

Neuroimaging studies provide converging evidence for these interactions at the systems level. Functional MRI studies demonstrate that sleep deprivation increases amygdala reactivity to emotional facial stimuli, particularly those conveying fear, while reducing functional connectivity between the amygdala and prefrontal regulatory regions, including the ventromedial PFC and anterior cingulate cortex (Motomura et al., 2013; Cheng et al., 2018). Similar patterns are observed in children, where shorter sleep duration is associated with increased amygdala responses to emotional faces and weakened emotion-dependent amygdala-cortical connectivity. Importantly, intranasal OT administration modulates amygdala responses to social stimuli, typically attenuating responses to fearful faces and enhancing responses to positive social cues, with effects varying by sex, dose, and amygdala subregion. The most robust attenuation of fear-related amygdala activity has been observed following administration of 24 IU intranasal OT approximately 45–70 min post-dose (Kirsch et al., 2005; Domes et al., 2007; Rilling et al., 2014).

Sleep deprivation is associated with increased social withdrawal and interpersonal distancing, as well as impaired social cognition, rather than broadly reduced social engagement (Ben Simon and Walker, 2018; Simon et al., 2022). These behavioral changes may reduce opportunities for social buffering during stress. Evidence linking OT signaling to these processes derives, at least in part, from studies of intranasal OT administration conducted in children with autism spectrum disorder (Alaerts et al., 2024). Thus, although OT-related pathways may modulate social and stress-responsive processes, extrapolation to endogenous OT signaling in neurotypical adults should be approached with caution, particularly given OT’s known affinity for the AVPR1A. This bidirectional relationship may generate a feedforward loop in which insufficient sleep weakens social connection and OT signaling, thereby increasing stress sensitivity and further disrupting sleep. Conversely, OT’s anxiolytic and pro-social effects may indirectly stabilize sleep by enhancing emotional security and social connectedness, factors known to buffer stress-related sleep disturbances.

Taken together, converging evidence indicates that sleep deficiency amplifies stress reactivity and impulsivity through dysregulation of the HPA axis and disruption of adaptive emotional and social networks (Minkel et al., 2014; Ben Simon and Walker, 2018; Simon et al., 2022). Within this framework, OT is well positioned to promote allostasis and resilience through its modulatory effects on stress, emotion, and social behavior. Future research should determine how OT mechanisms might be leveraged to mitigate the behavioral and neuroendocrine consequences of insufficient sleep, particularly in vulnerable populations (Figure 1).

## Clinical implications and future directions

OT has been extensively investigated for its potential to modulate mood, particularly in anxiety disorders, autism spectrum disorder (ASD), major depressive disorder (MDD) and other related affective conditions. Across these domains, intranasal OT has been explored primarily as a modulatory adjunct targeting social cognition and stress reactivity, rather than as a definitive anxiolytic or antidepressant.

In anxiety and mood disorders, findings remain mixed. For instance, a systematic review of 15 randomized controlled trials examining OT in anxiety and depressive disorders, including social anxiety, specific phobias, major depression, and postpartum depression, concluded that overall effects were inconsistent, with many studies limited by single-dose designs and small sample sizes (De Cagna et al., 2019). Although in a pilot double-blind randomized controlled trial, 23 adults with MDD received intranasal OT (24 IU) prior to each psychotherapy session and showed accelerated symptom improvement and strengthened early therapeutic alliance when compared with placebo (Ellenbogen et al., 2024). In contrast, other randomized controlled trials of intranasal OT in postpartum depression have found no significant improvements in depressive symptoms (Mah et al., 2013), highlighting the need for a more detailed mechanistic understanding of OT’s actions in the brain.

Intranasal OT has also been explored as a potential intervention for neurodevelopmental conditions such as ASD and for certain anxiety disorders for its potential to enhance social communication and affiliative processing. However, OT’s role in modulating sleep–wake regulation remains poorly characterized and requires further investigation (Raymond et al., 2021; Doerr et al., 2022). Despite growing interest, no OT-based therapies are approved to date for the treatment of insomnia, depression, ASD or anxiety, and clinical use remains experimental pending larger, well-controlled trials.

In healthy individuals, OT administration has been associated with reduced subjective stress ratings (Heinrichs et al., 2003) and decreased salivary cortisol concentrations (Ditzen et al., 2009). Increases in plasma OT have been observed following exposure to uncontrollable noise in women (Sanders et al., 1990) and in response to psychosocial stressors (Sanders et al., 1990). OT release during stress may help attenuate physiological stress responses, as higher basal plasma OT levels are associated with lower norepinephrine concentrations, reduced blood pressure, and decreased heart rate (Grewen et al., 2005). These findings suggest a role for OT in stress modulation, while translational implications for clinical sleep disturbances remain preliminary.

Importantly, OT’s behavioral effects are highly context-dependent and moderated by individual differences (Olff et al., 2013). When social cues are perceived as safe or affiliative, OT tends to promote prosocial behaviors and adaptive stress regulation. Conversely, when social cues are interpreted as threatening, OT may amplify defensive behaviors or heighten negative social perceptions. Clarifying these context-sensitive mechanisms will be essential for the rational development of pharmacological strategies targeting the OT system in neurodevelopmental, anxiety, and mood disorders (Rigney et al., 2022).

## Conclusion

Sleep, circadian rhythms, and stress regulation form an interdependent network that shapes social behavior, impulsivity, and cognition. Disruption of any one component can adversely affect the others, leading to widespread functional impairments. Within this network, oxytocin emerges as a potential neuromodulatory contributor, buffering stress responses, facilitating social bonding, and possibly contributing to the stabilization of sleep architecture. Emerging evidence, predominantly from preclinical and early translational studies, indicates that OT signaling may interact with sleep-related circuits to support emotional regulation, stress resilience, and memory consolidation. Nonetheless, additional human research is required to determine the extent and clinical significance of these effects, particularly within the proposed feedforward loop linking sleep loss, OT signaling, and stress sensitivity.
